# Utilising Network Pharmacology to Explore Underlying Mechanism of *Astragalus membranaceus* in Improving Sepsis-Induced Inflammatory Response by Regulating the Balance of I*κ*B*α* and NF-*κ*B in Rats

**DOI:** 10.1155/2022/7141767

**Published:** 2022-03-31

**Authors:** Haiyang Yu, Qihua Ling, Jingwen Cai, Mengzhi Zhang, Huaiquan Liu, Yunzhi Chen

**Affiliations:** ^1^Guizhou University of Traditional Chinese Medicine, Guiyang, Guizhou 550025, China; ^2^Shuguang Hospital Affiliated to Shanghai University of Traditional Chinese Medicine, Shanghai 201203, China; ^3^The Second Affiliated Hospital of Nanchang University, Nanchang, Jiangxi 330006, China

## Abstract

**Objective:**

The purpose of the present study was to explore the mechanism of *Astragalus membranaceus* in the treatment of sepsis.

**Methods:**

We searched the active components and targets of *Astragalus membranaceus* using the TCMSP and BATMAN databases. Then, the GeneCards, MalaCards, and OMIM databases were used to screen out relevant targets of sepsis. The common targets of the former two gene sets were uploaded to the STRING database to create an interaction network. DAVID was used to perform KEGG enrichment analysis of the core targets. Based on the results of KEGG and previous studies, key pathways for the development of sepsis were identified and experimentally validated.

**Result:**

We obtained 3,370 sepsis-related targets in databases and 59 active components in *Astragalus membranaceus* through data mining, corresponding to 1,130 targets. The intersection of the two types of targets led to a total of 318 common targets and 84 core targets were obtained after screening again. The KEGG and previous studies showed that these 84 core targets were involved in sepsis by regulating TNF, MAPK, and PI3K pathways. TNF, MAPK8, NF-*κ*B, and I*κ*B*α* are crucial in sepsis. Experimental validation demonstrated that some markers in sepsis model rats were improved after the intervention with *Astragalus* granules and their chemical components. Among them, IL-1*β*, IL-6, and TNF-*α* in rat serum were reduced. The mRNA and protein expression of TNF-*α*, IL-6, MMP9, MAPK8, and NF-*κ*B were reduced in rat blood. However, the mRNA and protein expression of I*κ*B*α* and PI3K were increased in rat blood.

**Conclusion:**

The AST could affect the TNF, PI3K, and MAPK pathway cascade responses centred on I*κ*B*α* and NF-*κ*B, attenuate the expression of IL-6 and MMP9, and interfere with the inflammatory response during sepsis.

## 1. Introduction

Sepsis is a systemic inflammatory disease caused by the overactivation of the immune system and a cascade of inflammatory molecules released in the body under stress conditions, such as infection by pathogenic microorganisms, trauma, or shock. Without timely and effective treatment, sepsis can develop into severe sepsis and threaten the patient's life [1, 2]. Epidemiology has shown that there are more than 30 million patients with sepsis worldwide [3]. Currently, the academic community suggests that excessive activation of the inflammatory response [4], immune dysfunction [5], and coagulation dysfunction [6] affect the prognosis of patients with sepsis. Clinically, the main treatment for sepsis is allopathic based on antibiotics. However, with the increasing number of drug-resistant strains, the abuse of antibacterial drugs, the development of new antibacterial drugs, and the relative lag in clinical application, the prognosis of patients with sepsis is still unsatisfactory [7].

*Astragalus membranaceus* (AST) is a Chinese herb that is effective in treating sepsis. AST is the dry root of *Astragalus membranaceus* (Fisch.) *Bge. var. mongholicus *(Bge.) Hsiao. modern pharmacological studies have shown that AST and its constituents have good pharmacological effects on organ damage caused by sepsis. Astragaloside IV attenuates sepsis-induced intestinal barrier dysfunction by inhibiting RhoA/NLRP3 inflammasome signal transduction [8]. *Astragalus* polysaccharides have a protective effect on septic heart dysfunction by inhibiting the TLR4/NF-*κ*B signaling pathway [9]. *Astragalus* saponins can also regulate the levels of serum myeloperoxidase, nitric oxide, and lactate dehydrogenase and reduce the mRNA expression of inducible nitric oxide synthase and interleukin-1*β* in the liver, alleviating sepsis caused by cecal ligation and puncture [10].

Network pharmacology is a discipline that uses network visualisation and other technologies to reveal the complex biological network relationship among drugs, genes, diseases, and targets [11]. Network pharmacology can analyse drugs acting on different targets, cells, and organs at the molecular and genetic levels as well as predict and reveal the action and mechanisms of drugs. Network pharmacology can be used to construct a drug-target network based on the structure and efficacy of drugs and effectively predict the medicinal components and mechanism of action of traditional Chinese medicines and their compound preparations [12]. Therefore, we explored the protective effect of AST on the body. This study used network pharmacology methods to predict the targets and pathways of multiple compounds in AST. By constructing a “compound-target-disease” network, we were able to further clarify the improvement mechanism of AST in the process of sepsis. At the same time, we intragastrically administered AST granules to SD rats for one week and then constructed a sepsis model by tail vein injection of LPS. Finally, crucial gene changes were detected to explore the protective effect of AST on sepsis.

## 2. Materials and Methods

### 2.1. The Chemical Composition of AST and Its Targets

The main chemical components of AST were retrieved from the Traditional Chinese Medicine Systems Pharmacology Database and Analysis Platform (TCMSP) (http://lsp.nwu.edu.cn/tcmsp.php) [13] and Bioinformatics Analysis Tool for Molecular mechANism of Traditional Chinese Medicine (BATMAN) [14] (http://bionet.ncpsb.org.cn/batman-tcm/). Based on the pharmacokinetic parameters of drug absorption, distribution, metabolism, and toxicity in the human body [15], oral bioavailability [16] (OB) ≥ 30% and druglikeness [17] (DL) ≥ 0.18 were used as the screening conditions for chemical components in TCMSP database. Adjusted *P* < 0.05 was used as the screening condition for chemical components in BATMAN database [18]. According to the screening conditions, the effective active components of AST were initially screened. If the screened composition has no target, then we delete it.

### 2.2. Search for Sepsis-Related Targets

The Online Mendelian Inheritance in Man (OMIM, https://www.omim.org/) and the Human Disease Database (MalaCards, https://www.malacards.org/), and the Human Gene Database (GeneCards, https://www.genecards.org/) were the searched target genes using “sepsis” as a keyword. After taking intersections of sepsis-related targets with AST targets, we obtained potential genes for AST in the treatment of sepsis.

### 2.3. Protein-Protein Interaction (PPI) Construction and Screening

The intersecting genes were imported into the STRING database 11.5 (http://string.embl.de/). Species was set as human; the minimum interaction threshold was set to “highest confidence” (>0.9) [19]; and the disconnected nodes in the network were hidden. In addition, a secondary screen was performed to obtain more accurate targets. The screening criteria were as follows: targets with a degree, betweenness, and closeness greater than the median value of all the nodes in the intersection network were selected as core targets to build the protein-protein interaction (PPI) network [20].

### 2.4. Construction of the Drug-Target-Disease Network Graph

We imported the core genes and their corresponding components into Cytoscape 3.6.1 in order to construct the Drug-Target-Disease Network graph.

### 2.5. Kyoto Encyclopedia of Genes and Genomes (KEGG) Enrichment Analysis

To further understand the specific roles of the screened intersection networks in terms of gene function and related signaling pathways, KEGG pathway enrichment analysis was performed on the genes associated with the core targets using DAVID. A threshold of *P* < 0.05 was set as statistically significant. Potential connections between pathways were also found by searching previous literature in PubMed, and the essential targets of the significant pathways were visualised using Cytoscape 3.6.1.

### 2.6. Molecular Docking

Molecular docking was performed to validate the important targets screened by KEGG analysis as well as their more closely related chemically synthesised components. Protein targets were obtained from the RCSB PDB (https://www.rcsb.org/) database, and compounds were obtained from the TCMSP database. The proteins and small molecules were optimised using SYBYL-X.2.0 software, and molecular docking was performed using the SurflexDock module. The interaction of the active ingredients with the target proteins was scored according to the total score scoring function, with larger total score values indicating better matching of the small molecule compound to the more significant protein. Generally, a score above 4.0 is considered to indicate some binding activity, and a value greater than 5.0 indicates that the molecule has strong binding to the target [21, 22].

### 2.7. Experimental Validation

#### 2.7.1. Main Reagents and Instruments

We used the following drugs and reagents: cycloeucalenol, encecalin, kaempferol (Chengdu Push Bio-Technology Co., Ltd, PS210726-09, PS210726-08, and PS011676), Interleukin-1*β* (IL-1*β*), Interleukin-6 (IL-6), Tumor Necrosis Factor (TNF-*α*) enzyme-linked immunoassay (ELISA) kits (Wuhan Genome Biotechnology Co., Ltd., JYM0419Ra, JYM0646Ra, and JYM0635Ra), LPS (Solarbio, L8880), rabbit polyclonal antibodies TNF-*α*, MAPK8, PI3K, IL-6 (Affinity, No. AF7014, DF6089, AF6241, and DF6087), GAPDH (Hangzhou Xianzhi Biological Co., Ltd., No. AB-P-R 001), rabbit monoclonal antibody I*κ*Ba, MMP9, NF-*κ*B, and *β* Actin (abcam No. Ab32518, Ab76003, Ab32360 and Ab6276), Methanol (Merck, Germany), *Astragalus* granules were purchased from Baili Pharmaceutical Group in China (authorised document number: country medicine accurate character Z20003380), excipient (Referring to Liang Jun's production process [23], the auxiliaries were made in Guizhou University of Traditional Chinese Medicine), TRIzol (Ambion, 15596-026), HiScript Reverse Transcriptase (VAZYME, R101-01/02), 5X HiScript Buffer (VAZYME, R101-01/02), ddH2O (genecopoeia, C1D230A), Ribonuclease Inhibitor (TransGen, AI101), dNTPs (TIANGEN, CD117), SYBR Green Master Mix (VAZYME, Q111-02), Taq Plus DNA Polymerase (TIANGEN, ET105-01), DL2000 DNA Marker (TIANGEN, MD114-02), and Random Primer (TAKARA, 3801); Primers (Wuhan Tsingke Biological Company, China).

We used the following instruments: HBS-1096A ELISA Analytical Instrument (Thermo Electron Corporation, America), adjustable micropipette (Glison, France), high-speed and low-temperature centrifuge (Eppendorf, Germany), low-speed centrifuge (Shanghai Anting Technology Instrument Factory, China), thermostat (Beijing Liuyi Company, China), 4°C refrigerators (Zhongke Meiling, China), 37°C incubation box (Henan Jinbo, China), and QuantStudio 6 real-time quantitative PCR instrument (ABI, America); High-Performance Liquid Chromatography (waters, America) and PCR instrument (Dongsheng Innovative Biotechnology Co., Ltd., China).

#### 2.7.2. Determining Chemical Compounds and LD50 Prediction Based on HPLC and Discovery Studio

Dissolve 4 g of *Astragalus* granules in 10 mL of ultrapure water, add methanol to fix the volume to 50 mL, extract with ultrasound for 2 hours, and then place in a centrifuge at 4°C and centrifuge at 12000 rpm for 10 min. 1 mg standard product of cycloeucalenol is dissolved in 1 mL methanol. 1 mg standard product of encecalin is dissolved in 1 mL methanol. 1 mg standard product of kaempferol is dissolved in 1 mL methanol.

For the detection of cycloeucalenol, a Waters model 2695 HPLC with a Waters 2424 evaporative light detector was used. The chromatographic column is Diamonsil C18 (2) (250 *∗* 4.6 mm, 5 *μ*m), the mobile phase is methanol, column temperature is 60°C, the flow rate is 1 mL/min, the drift tube temperature is 80°C, nebuliser temperature is 30°C, the nitrogen flow rate and gain is set to 25 psi and 100, respectively, and the injection volume is 10 *μ*L.

Encecalin was detected using a Waters Model 2695 HPLC with a DAD detector. The chromatographic column is Diamonsil C18 (2) (250 *∗* 4.6 mm, 5 *μ*m), the flow rate is 1 mL/min, the column temperature is 35°C, the detection wavelength is 254 nm, and the injection volume is 10 *μ*L. The gradient elution process is shown in Table 1.

The instrument used for the determination of kaempferol was a Waters model 2695 HPLC with DAD detector. The chromatographic column was a Diamonsil C18 (2) (250 *∗* 4.6 mm, 5 *μ*m) with a flow rate of 1 mL/min, a column temperature of 35°C, and a detection wavelength of 360 nm. The mobile phase was methanol : 0.1% phosphoric acid in water/50 : 50, and the injection volume was 10 *μ*L.

Oral LD50 predictions for cycloeucalenol, encecalin, and kaempferol were performed in rats using Discovery Studio software to determine the subsequent dose to be administered [24], with 1/20th of the respective LD50 resultant dose being used as the final dose for subsequent pharmacological experiments [25].

#### 2.7.3. Experimental Animals

Seventy SD male rats aged 8∼9 weeks with bodyweight of (200 ± 30) g were used in this laboratory, all of which were provided by Changsha Tianqin Biotechnology Co. LTD. (animal production licence number: SCXK (Xiang) 2019-0014). According to the random number table method, they were randomly divided into 7 groups with 10 rats in each group, namely: control group, LPS group, LPS + *Astragalus* granules group, LPS + excipient group, LPS + cycloeucalenol group, LPS + encecalin group, and LPS + kaempferol group. The normal adult dosage of *Astragalus* granule is 8g per day. Based on the human-rat body surface area conversion, the daily dose for rats is 0.8 g/kg. Dissolve 8 g of *Astragalus* granules in 100 ml of water to prepare an aqueous solution of *Astragalus* granules at 80 mg/ml. This study was approved by the Medical Research Ethics Committee of the Second Affiliated Hospital of Nanchang University and the examination and approval no. review [2021], no. (A801).

#### 2.7.4. Sepsis Model Preparation and Sampling

After a week of adaptive feeding, each group of rats was given the appropriate intervention. *Astragalus* granule (0.8 g/kg), excipient (0.8 g/kg), cycloeucalenol (350 mg/kg), encecalin (14.1 mg/kg), and kaempferol (6.9 mg/kg), respectively, were administered by gavage for one week. LPS modelling was performed on all rats except control group 24 h after the last administration. In the control group, only normal saline was injected into the tail vein. Rats in other groups were injected with 10 mg/kg of LPS through tail vein according to body weight [26]. Twenty-four hours after model establishment, all surviving rats in each group were anaesthetised for blood sampling.

#### 2.7.5. Observing the General Situation and Survival Rate of Rats

Behavior change, fur color, and mental state before and after modelling were observed. Death after modelling was recorded, and survival curves of rats in each group were drawn.

#### 2.7.6. Detection of Serum IL1*β*, IL-6, and TNF-*α* Levels in Rats by ELISA

After anaesthesia, abdominal aorta blood was taken and centrifuged. The supernatant was taken, and the contents of IL1*β*, IL-6, and TNF-*α* in serum were detected by ELISA kit. The operation was carried out in strict accordance with the instructions.

#### 2.7.7. Real-Time Quantitative PCR Analysis

Total RNA was extracted from rat blood using a TRIzol kit (Takara) according to the manufacturer's instructions, reverse transcribed into cDNA using the PrimeScript RT kit with genomic decontamination, and then amplified by PCR. GAPDH was used as an internal reference gene to calculate the 2^-ΔΔCt^ value for IL-6, MAPK8, TNF-*α*, MMP9, PI3K, and NF-*κ*B genes. *β*-actin was used as an internal reference gene to calculate the 2^-ΔΔCt^ value for I*κ*B*α* gene. The specific primers were as follows (as shown in Table 2).

#### 2.7.8. Western Blot

Use RIPA lysate to lyse the cells in the blood, use the BCA kit to determine the protein concentration, add the prepared protein sample to a 10% gel for electrophoresis, transfer the membrane, and then block it in 5% skim milk for 2 hours. PVDF membranes of 0.45 *μ*m size were immersed in primary antibody incubation solution and incubated overnight at 4°C. Antibody dilutions were as follows: GAPDH (1 : 1000), TNF (1 : 500), *β*-actin (1 : 200), MAPK8 (1 : 1000), PI3K (1 : 2000), NF-*κ*B (1 : 1000), IL-6 (1 : 1000), I*κ*Ba (1 : 5000), and MMP9 (1 : 5000). Add the corresponding secondary antibody, soak the PVDF membrane in the secondary antibody incubation solution, and incubate for 2 h at 37°C in a shaker. Finally, the film was washed with TBST and developed with ECL luminescence. The films were scanned, and the results were analysed by ImageJ software for gel imaging systems.

#### 2.7.9. Statistical Methods

All data were processed by SPSS 22.0 statistical software. The measured data are expressed as the mean ± standard deviation (mean ± SD). One-way ANOVA was used to compare multiple groups when the data were normally distributed and homogeneity of variance. Survival analysis was performed using the log-rank (Mantel–Cox) test function. Differences were indicated as statistically significant at *P* < 0.05.

## 3. Results and Discussion

### 3.1. Results

#### 3.1.1. AST Chemical Composition and Its Targets

After screening, the TCMSP database contains 8 compounds and 111 targets, and the BATMAN database contains 51 compounds and 1081 targets. After removing the blank and duplicate targets, a total of 59 chemical components and 1,130 drug targets were obtained (as shown in Table 3).

#### 3.1.2. Potential Targets of AST for Sepsis

**Figure 1:**
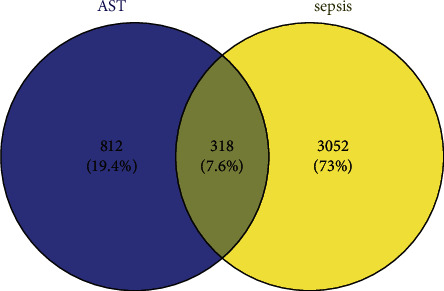
Intersecting genes of AST and sepsis. The blue circle on the left represents the target of AST. The yellow circles at the right represent sepsis targets. The intersection in the middle indicates the potential target of AST for the treatment of sepsis.

2498, 771, and 183 targets associated with sepsis were obtained from GeneCards, MalaCards, and OMIM, respectively. A total of 3370 pathogenic genes were obtained after the removal of duplicates for these targets. A total of 318 intersecting genes were obtained after intersecting these pathogenic genes with the 1130 targets of AST. These are potential targets that can be used in the treatment of sepsis (as shown in Figure 1).

#### 3.1.3. Protein-Protein Interaction (PPI) Network Construction and Screening

**Figure 2:**
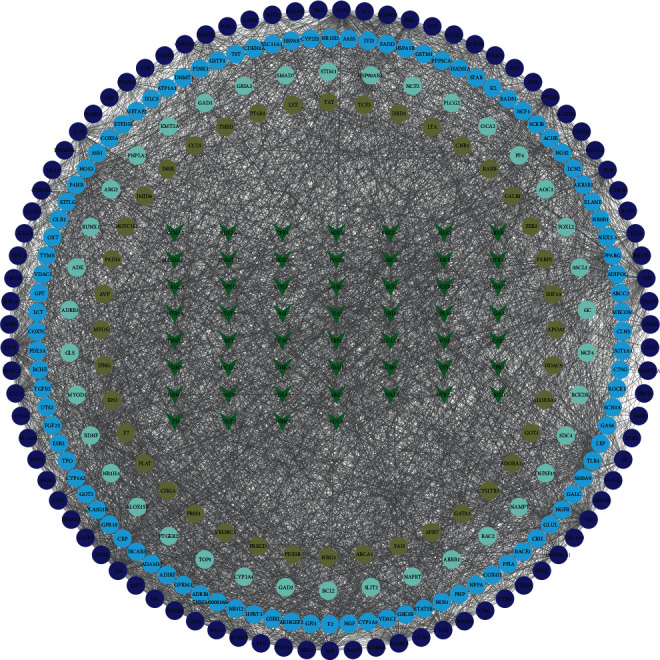
Protein-protein interaction of 318 genes is shown.

To further clarify the extent of the role of intersecting genes in the development of sepsis, we screened 318 intersection-based genes by the STRING database. Based on the magnitude of the median degree, betweenness, and closeness of individual genes in the entire network, we identified 84 core targets (as shown in Figure 2).

#### 3.1.4. AST Active Ingredient Target-of-Action Disease Network Diagram

**Figure 3:**
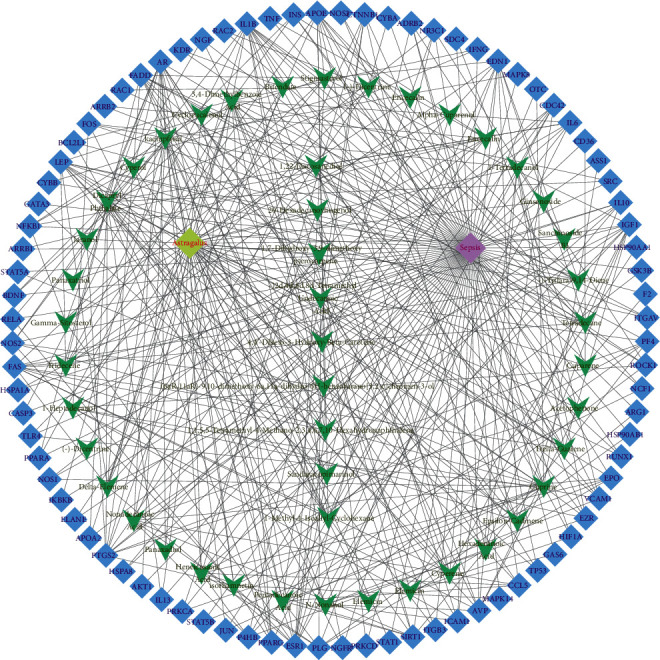
AST active ingredient target-of-action disease network diagram. Components of the drugs are shown as green shuttles, and the 84 core targets of the components acting on the disease are marked with blue squares and arranged in outer circles. Inside the circles, the yellow and purple squares represent “AST” and “sepsis,” respectively.

The 84 core targets and their corresponding 44 chemo-components were visualised using Cytoscape 3.6.1 software (as shown in Figure 3).

#### 3.1.5. KEGG Result Presentation

**Figure 4:**
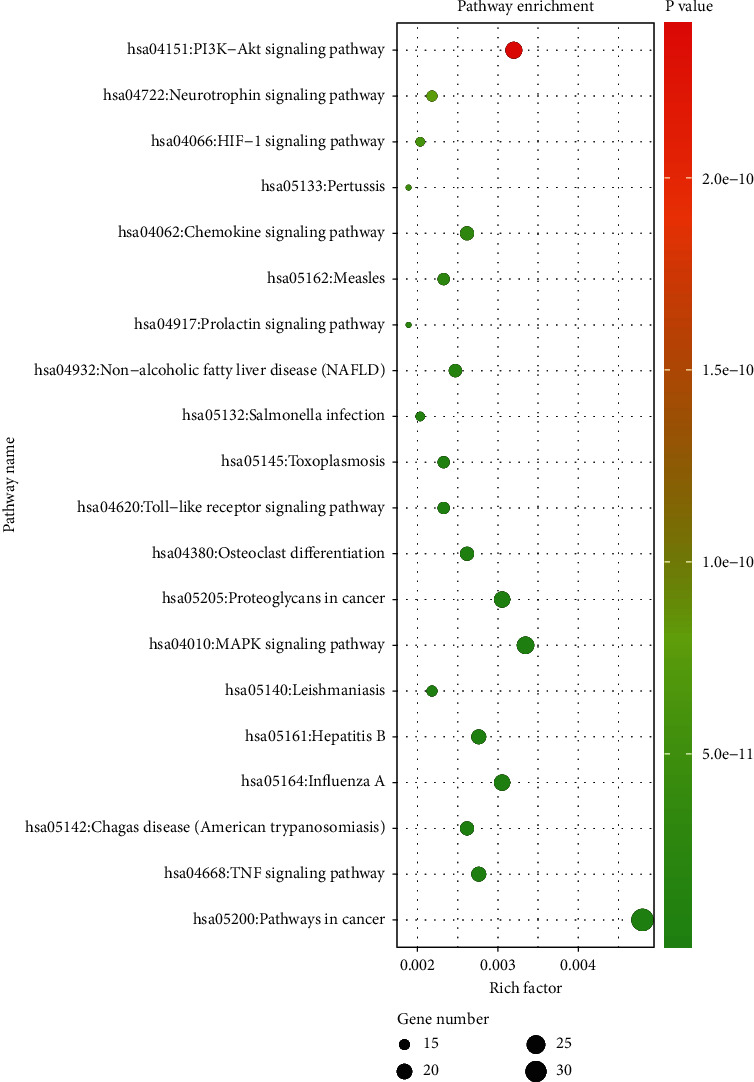
KEGG bubble chart. The size of dots indicates the number of genes. The color of dots indicates the size of *P* value.

**Figure 5:**
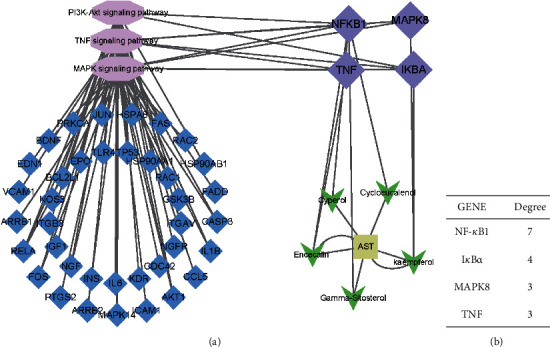
Network diagram of core targets, pathways, and components. (a) Network diagram of core targets, pathways, and components. (b) Degree of core genes in the network diagram.

KEGG analysis of the targets related to the treatment of sepsis by AST was performed using DAVID. Eighty-four markers were found to be involved in 104 signaling pathways (*P* < 0.05), mainly including cancer, the TNF signaling pathway, Chagas disease, influenza A, hepatitis B, leishmaniasis, the MAPK signaling pathway, proteoglycans in cancer, osteoclast differentiation, Tollas disease, osteoclast differentiation, influenza A, hepatitis B, leishmaniasis, the MAPK signaling pathway, proteoglycans in cancer, osteoclast differentiation, the Toll-like receptor signaling pathway, toxoplasmosis, Salmonella infection, nonalcoholic fatty liver disease (NAFLD), the prolactin signaling pathway, measles, the chemokine signaling pathway, pertussis, the HIF-1 signaling pathway, neurotrophin signaling pathway, the PI3K-Akt signaling pathway, etc. In this study, the top 20 KEGG signaling pathways were screened according to the count value to generate a map (as shown in Figure 4). Based on the KEGG results and summary of previous studied, we found the TNF, MAPK, and PI3K pathways in the pathogenesis of sepsis [27]. We finally identified I*κ*B*α*, MAPK8, NF*κ*B1, and TNF as possible indicators of sepsis development based on the frequency of 84 core targets in the TNF, MAPK, and PI3K pathways and relevant references [28–30]. In Figure 3, the compounds corresponding to these targets, include kaempferol, cycloeucalenol, cyperol, encecalin, gamma-sitosterol, and encecalin, may play an important therapeutic role. We identified the active components in the AST based on the core targets and mapped the network diagram of core targets, pathways, and components in conjunction with the KEGG results (as shown in Figure 5).

#### 3.1.6. Molecular Docking

**Figure 6:**
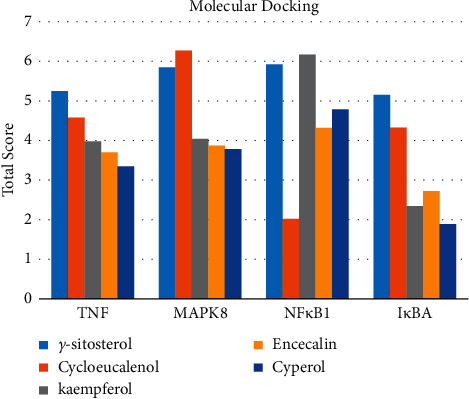
Molecular docking total score. The horizontal axis indicates the protein. The vertical axis indicates the score of the compound to protein docking results.

**Figure 7:**
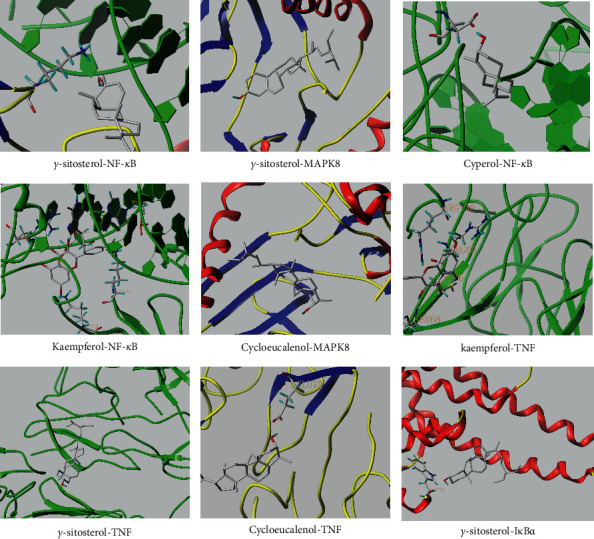
Molecular docking. Ribbons represent proteins. The molecule directly attached to the ribbon is the ligand. The molecule not directly linked to the strip is a compound of AST.

The main active components of AST obtained in Section 3.1.5 were *γ*-sitosterol, cycloeucalenol, kaempferol, encecalin, and cyperol. TNF, MAPK8 (JNK), NF-*κ*B1, and I*κ*B*α* were the critical genes identified in Section 3.1.5. The protein receptors and active compounds corresponding to I*κ*B*α* (PID: 6Y1J), NF-*κ*B (PID: 1NFK), TNF (PID: 1DU3), and MAPK8 (PID: 4G1W) were selected from the RSCB PDB database for molecular docking. The main active components in AST enter the active site during the docking process with the core targets, indicating that the main active components and the core targets demonstrate good binding, which verifies the reliability of the results of this study. The total score results are shown in Figure 6. Graph showing molecular docking results is given in Figure 7.

#### 3.1.7. Experimental Validation

**Figure 8:**
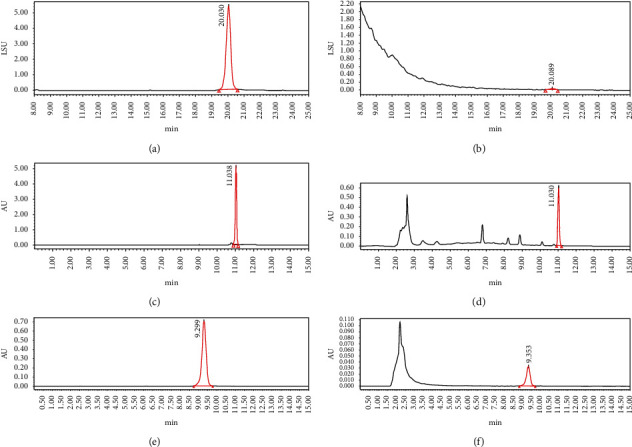
HPLC chromatogram. (a) Cycloeucalenol standard chromatogram. (b) Cycloeucalenol chromatogram in *Astragalus* granules. (c) Encecalin standard chromatogram. (d) Encecalin chromatogram in *Astragalus* granules. (e) Kaempferol standard chromatogram. (f) Kaempferol chromatogram in *Astragalus* granules.

*(1) Determination of Substances and Prediction of LD50 Based on HPLC and Discovery Studio for Chemistry Components*. In chromatographic analysis, samples of *Astragalus* granules showed the same retention times as the standard products of cycloeucalenol, encecalin, and kaempferol (as shown in Figure 8).

According to the calculation by Discovery Studio, the oral LD50 of cycloeucalenol, encecalin, and kaempferol was 7 g/kg, 282.7 mg/kg, and 138.9 mg/kg, respectively.

**Figure 9:**
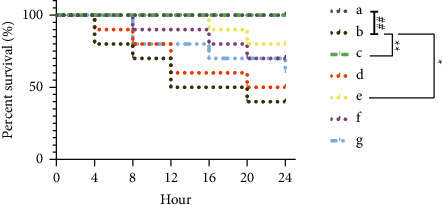
Effect of *Astragalus* granules and its components on the survival rate of rats with sepsis (*n* = 10). a: Control group; b: LPS group; c: LPS + *Astragalus* granules group; d: LPS + excipient group; e: LPS + cycloeucalenol group; f: LPS + Encecalin group; g: LPS + Kaempferol group. ^∗^*P* <0.05, compared with the LPS group. ^∗∗^*P* <0.01, compared with LPS group. ^##^*P* < 0.01, compared with control group.

*(2) General Situation and Survival Analyses*. Compared with the control group, rats in the LPS group, LPS + *Astragalus* granules group, LPS + excipient group, LPS + cycloeucalenol group, LPS + encecalin group, and LPS + kaempferol group have decreased diet and water consumption. LPS group and LPS + excipient group have the worst mental state and weakened activities, while LPS + *Astragalus* granules group and LPS + cycloeucalenol group situation was significantly improved. When the time to death of the rats within 24 hours was counted, a significant difference was found in the survival curve of LPS group compared to control group (*P* < 0.01). This demonstrated that LPS has a strong lethal effect. The survival rate was significantly improved with the intervention of *Astragalus* granules and cycloeucalenol (*P* < 0.05) (as shown in Figure 9). This implied that *Astragalus* granules and cycloeucalenol could significantly reduce the toxicity of LPS in rats.

**Figure 10:**
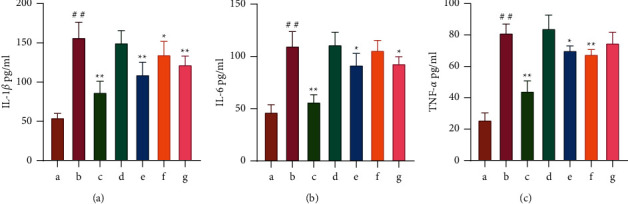
Effects of *Astragalus* granules and its components on the levels of IL-1*β*, IL-6, and TNF-*α* in serum of rats in each group (*n* = 4). (a) Expression of IL-1*β* in the serum of rats in each group; (b) Expression of IL-6 in the serum of rats in each group, (c) Expression of TNF-*α* in the serum of rats in each group. a: Control group; b: LPS group, c: LPS + *Astragalus* granules group; d: LPS + excipient group; e: LPS + cycloeucalenol group; f: LPS + Encecalin group; g: LPS + Kaempferol group. ^∗^*P* <0.05, compared with LPS group. ^∗∗^*P* <0.01, compared with LPS group. ^##^*P* < 0.01, compared with control group.

*(3) Determination of the Serum IL-1β, IL-6, and TNF-α Levels in Rats by ELISA*. IL-1*β*, IL-6, and TNF-*α* are critical inflammatory factors and biological markers of sepsis [31]. They can be used as a criterion for the success of the sepsis model. This study verified the expression of serum inflammatory factors in vivo in septicemic rats affected by sepsis. IL-1*β*, IL-6, and TNF-*α* of serum expression of biological markers were increased in the LPS group compared to that in the control group (*P* < 0.01). The levels of IL-1*β*, IL-6, and TNF-*α* were downregulated after *Astragalus* granules intervention (*P* < 0.01). Cycloeucalenol significantly inhibited IL-1*β* (*P* < 0.01) and also suppressed the elevation of IL-6 and TNF-*α* to some extent (*P* < 0.05). Encecalin significantly inhibited TNF-*α* (*P* < 0.01) and also suppressed the elevation of IL-1*β* to some extent (*P* < 0.05). Kaempferol significantly inhibited IL-1*β* (*P* < 0.01) and also suppressed the elevation of IL-6 to some extent (*P* < 0.05) (as shown in Figure 10).

**Figure 11:**
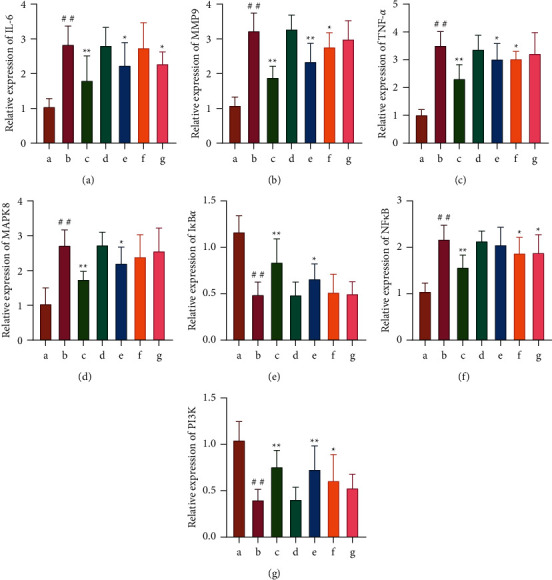
PCR results (*n* = 3). (a–g) Effects of *Astragalus* granules and their components on the expression levels of IL-6, MMP9, TNF-*α*, MAPK8, I*κ*B*α*, NF-*κ*B, and PI3K mRNA in the blood of rats in each group. a: control group; b: LPS group; c: LPS + *Astragalus* granules group; d: LPS + excipient group; e: LPS + cycloeucalenol group; f: LPS + encecalin group; g: LPS + kaempferol group. ^∗^*P* <0.05, compared with LPS group. ^∗∗^*P* <0.01, compared with LPS group. ^##^*P* < 0.01, compared with control group.

*(4) Detection of TNF-α, IL-6, MMP9, MAPK8, PI3K, NF-κB, and IκBα mRNA Expression in Rat Blood by Real-Time qPCR*. The result showed that the targets of TNF-*α*, IL-6, MMP9, MAPK8 (JNK), and NF-*κ*B in the blood of rats in the LPS group were significantly higher than those in the blood of rats in the control group (*P* < 0.01), and PI3K and I*κ*B*α* were substantially lower than those in the blood of rats in the control group (*P* < 0.01). This trend was reversed after *Astragalus* granules intervention (*P* < 0.01). In addition, cycloeucalenol, encecalin, and kaempferol also regulate the expression of these genes in the blood of the sepsis model to varying degrees. Among them, cycloeucalenol significantly inhibited the mRNA expression of MMP9 (*P* < 0.01) and the mRNA expression of inhibited MAPK8, TNF-*α*, and IL-6 (*P* < 0.05). Cycloeucalenol significantly upregulated PI3K expression (*P* < 0.01) and I*κ*B*α* (*P* < 0.05). Encecalin inhibited the upregulation of MMP9, NF-*κ*B, and TNF-*α* (*P* < 0.05). Encecalin upregulated PI3K expression (*P* < 0.05). Kaempferol inhibited the elevation of NF-*κ*B and IL-6 (*P* < 0.05). These manifestations illustrate that *Astragalus* granules and their components can regulate the expression of these genes during transcription (as shown in Figure 11).

**Figure 12:**
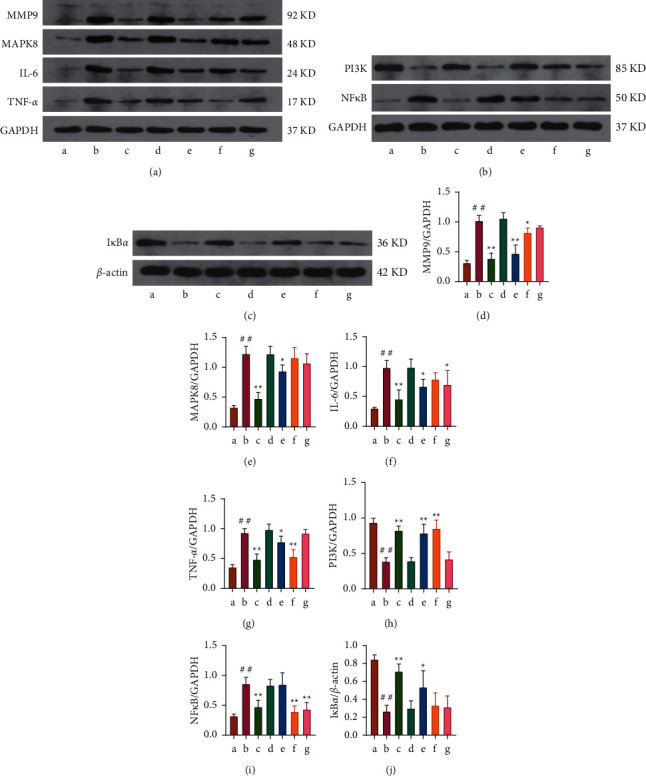
The protein expression levels of TNF-*α*, IL-6, MMP9, MAPK8, PI3K, NF-*κ*B, and I*κ*B*α* in blood (*n* = 3). (a) Western blot analysis of the proteins MMP9, MAPK8, IL-6, and TNF-*α* in the blood. (b) Western blot analysis of the proteins PI3K and NF-*κ*B in the blood. (c) Western blot analysis of the proteins I*κ*B*α* in the blood. (d–j) Protein expression of TNF-*α*, IL-6, MMP9, MAPK8, PI3K, NF-*κ*B, and I*κ*B*α*. a: control group; b: LPS group; c: LPS + *Astragalus* granules group; d: LPS + excipient group; e: LPS + cycloeucalenol group; f: LPS + encecalin group; g: LPS + kaempferol group. ^∗^*P* <0.05, compared with LPS group. ^∗∗^*P* <0.01, compared with LPS group. ^##^*P* < 0.01, compared with control group.

*(5) The Protein Expression Levels of TNF-α, IL-6, MMP9, MAPK8, PI3K, NF-κB, and IκBα in Rat Blood*. Our study revealed significant changes in blood protein expression of core targets such as TNF-*α*, IL-6, MMP9, MAPK8, PI3K, NF-*κ*B, and I*κ*B*α* in LPS group after LPS intervention (*P* < 0.01). The protein expression of TNF-*α*, IL-6, MMP9, MAPK8, and NF-*κ*B was significantly increased in the LPS group (*P* < 0.01), while the protein expression of PI3K and I*κ*B*α* was significantly decreased (*P* < 0.01). These trends were significantly reversed in LPS + *Astragalus* granules group after intervention with *Astragalus* granules (*P* < 0.01). In addition, cycloeucalenol, encecalin, and kaempferol can also regulate the expression of related proteins in the blood of the sepsis model to varying degrees. Among them, cycloeucalenol significantly inhibited the expression of MMP9 (*P* < 0.01) and inhibited MAPK8, TNF-*α*, and IL-6 (*P* < 0.05). Cycloeucalenol significantly upregulated PI3K expression (*P* < 0.01) and I*κ*B*α* (*P* < 0.05). Encecalin significantly inhibited the upregulation of MMP9 (*P* < 0.05), TNF-*α*, and NF-*κ*B (*P* < 0.01). Encecalin significantly upregulated PI3K expression (*P* < 0.01). Kaempferol significantly inhibited NF-*κ*B (*P* < 0.01) and IL-6 (*P* < 0.05) (as shown in Figure 12).

### 3.2. Discussion

As an inflammatory blood disease, the occurrence and development of sepsis is the result of multiple protein molecules and multiple signal pathways modulating on various cell biological behaviors. AST, a traditional Chinese medicine, has a significant effect on sepsis, and it can play a role in the treatment of sepsis in various ways. Recent studies have shown that there are many components in the AST that can slow the progression of sepsis. The literature has demonstrated that kaempferol, stigmasterol, (-)-dicentrine, 1-tetradecanol, cyperene, tetradecane, tridecene, elemicin, and other components can relieve the inflammatory response of the body. Stigmasterol can effectively inhibit the inflammatory response induced by LPS and has an excellent regulatory impact on the abnormal levels of serum liver enzyme markers, alanine aminotransferase, aspartate aminotransferase, ALT, and AST [32]. Kaempferol can be used to treat acute and chronic inflammatory damage to many organs, such as the colon, liver, and lung [33]. (-)-Dicentrine can inhibit the MAPK/Akt pathway activated by LPS to a certain extent and relieve the body's inflammatory response [34]. The tetradecane family can affect PPAR*γ* signaling and weaken tissue damage in the intestine [35]. Cyperene has an excellent protective effect on LPS-induced inflammation and oxidative stress damage in astrocytes [36]. Elemicin and other components have significant effects on inhibiting pneumonia, reducing serum IFN-*γ* and IL-4 levels, and enhancing their antioxidant activity [37]. *γ*-sitosterol can inhibit the expression of NF-*κ*B and the synthesis of TNF-*α* in macrophages [38]. AST can alleviate inflammatory damage in patients with sepsis by regulating the MAPK pathway, PPAR*γ* signaling, IFN-*γ*, and IL-4.

After analysing the PPI network, it was found that TNF, MAPK14, AKT1, MAPK8 (JNK), and NF-*κ*B1 were important in the progression of the disease. TNF is a bipolar molecule derived from macrophages and immune cells. When the body is activated during infection or tissue damage, it can transmit signals via ligands and receptors back to cells, causing subsequent inflammatory cascade reactions [39]. TNF-*α* can not only induce a massive release of a variety of inflammatory factors and stimulate the development of inflammation [40] but also directly consume the antioxidant substance glutathione in the body [41] and stimulate neutrophils and endothelial cells to release oxygen free radicals and other free radicals [42]. MAPK14 (p38) and MAPK8 (JNK) can receive and prolong the path and duration of TNF signal transduction and jointly affect the level of systemic inflammation response, the area of tissue damage, and the degree of organ function impairment by activating NF-*κ*B [43]. In this process, AKT1 (also called protein kinase B or PKB) activates I*κ*B*α* under the action of PI3k. I*κ*B*α* can pass through the 300 amino acid residue Rel homology region. The Rel homology domain (RHD) interacts with I*κ*B*α* to prevent the NF-*κ*B dimer from entering the nucleus to exert its negative feedback regulation on NF-*κ*B [44, 45]. In summary, the above targets will eventually activate the PI3k, MAPK, TNF, and NF-*κ*B pathways to aggravate the inflammatory response in the body.

According to the KEGG results and previous literature, we found that the PI3K signaling pathway, MAPK signaling pathway, and TNF signaling pathway played essential roles in the pathogenesis of sepsis. The PI3K (phosphatidylinositol-3-kinase) pathway is a crucial signaling pathway in the body, and it maintains tissue homeostasis by regulating protein phosphorylation, methylation, and ubiquitination [44]. In sepsis, activation of the PI3K pathway can reduce damage to the diaphragm [46], myocardium [47], lung [48], and other tissues and organs by inhibiting the degradation of I*κ*B*α*. Mitogen-activated protein kinase (MAPK) is an essential member of the intracellular signaling protein network that mediates extracellular stimuli to intracellular responses. The activation of its subtype JNK is based on thermal-induced apoptosis of macrophages, and the release of IL-6, MMP9, TNF-*α*, and other inflammatory factors further aggravates organ damage in patients with sepsis [49]. These inflammatory mediators in blood circulation mediate the damage to host cells, tissues, and organs and act as triggers of subsequent cascade reactions [50]. IL-6 is an essential indicator for evaluating the degree of the inflammatory response [51], and it can interact with a variety of cytokines, initiate a series of signal transduction mechanisms, and form a complex network of cytokines, which can mediate endothelial cells and monocytes [52]. The production of chemotactic protein (MCP)-1 can also recruit peripheral blood mononuclear cells to accumulate at the site of inflammation, activate specific white blood cells, and initiate and maintain an inflammatory response [53]. TNF-*α* can effectively control the increase in MMP9 expression after initiation by MAPK8 (JNK) [54], and the increase in MMP9 can promote a further inflammatory response and degrade extracellular matrix components [55]. In addition, TNF-*α* in combination with TNFR leads to the high expression of the transcription factor NF-*κ*B and aggravates the inflammatory response throughout the body [56]. Under normal circumstances, the combination of the NF-*κ*B dimer and I*κ*B*α* in resting cells masks the nuclear localisation signal of NF-*κ*B, but when stimulated, I*κ*B*α* is degraded, so the NF-*κ*B dimer is released into the nucleus and activates the transcription of target genes [57]. After NF-*κ*B is released, it initiates MAPK8 (JNK) and aggravates the inflammatory response [58]. The PI3K pathway is critical for synthesising I*κ*B*α* [59, 60]. Therefore, these three factors do not independently affect the physiological and pathological changes of the body. During the development of sepsis, these pathways are mostly interrelated and intertwined. The TNF signaling pathway is an essential condition for the early activation of the MAPK and PI3K signaling pathways, and it also establishes a connection between the PI3K and MAPK signaling pathways in the inflammatory response by regulating the hub of the NF-*κ*B pathway. The TNF, MAPK, and PI3K signaling pathways are connected through I*κ*B*α* and NF-*κ*B. The interaction between these pathways in the pathogenesis of sepsis determines the degree of widespread inflammation throughout the body, and exchange among these three pathways may be achieved by affecting the balance of I*κ*B*α* and NF-*κ*B.

Molecular docking technology is an essential means to confirm the interaction between a compound and its target [61]. To verify whether the critical targets in the above three pathways can be combined with the internal chemical components of AST, we used SYBYL-X.2.0 to carry out molecular docking between markers and compounds that are closely related to these pathways that had a total score greater than 4, which is a sensible standard for defining the combination of the markers and compounds. The results showed that *γ*-sitosterol, cycloeucalenol, kaempferol, encecalin, and cyperol bound well to the joint targets of TNF, MAPK8 (JNK), NF-*κ*B, and I*κ*B*α* in these three pathways. However, although we can determine the potential of binding between a compound and its target through this method, the biological changes of the target gene after binding are still unclear.

Because the interactions that occur among the chemical components of *Astragalus* granules are not clear, the advantage of Chinese medicine lies in the superimposed effect of multiple features on multiple pathways. Core genes such as TNF-*α*, MAPK8 (JNK), NF-*κ*B, and I*κ*B*α* were validated by ELISA, real-time quantitative PCR, and western blot. The results showed that the levels of sepsis markers IL1*β*, IL-6, and TNF*α* in the blood of model rats and the mortality rate of rats were significantly increased after tail vein injection. These phenomena are closely related to alterations in the PI3K signaling pathway, MAPK signaling pathway, and TNF signaling pathway in rats. The high mortality of rats is often accompanied by an imbalance between their own internal NF-*κ*B and I*κ*B*α*.The balance between NF-*κ*B and I*κ*B*α* can be altered by the expression of key genes such as TNF-*α*, IL-6, MMP9, MAPK8, and PI3K. Fortunately, *Astragalus* granules and their components significantly modulated mortality and altered the expression of these key genes in rats.

Indeed, the effects of cycloeucalenol, encecalin, and kaempferol are somewhat deviated compared to the effects of *Astragalus* granules. These compounds exhibit some targeting of the regulation of these key genes. For example, although cycloeucalenol has a modulating effect on most indicators, it has a weaker modulating effect on NF-*κ*B. Encecalin can modulation of MMP9, TNF-*α*, PI3K, and NF-*κ*B. Kaempferol is more likely to affect targets such as IL-6 and NF-*κ*B. Therefore, to some extent, cycloeucalenol, encecalin, and kaempferol can all reduce the expression of inflammatory factors and reduce the mortality rate in rats.

This demonstrates that the pharmacological action of *Astragalus* granules may be the result of the combined action of its internal chemotactic components such as cycloeucalenol, encecalin, and kaempferol. The pharmacological effects of *Astragalus* granules cannot be replaced by their own internal chemotactic components. Of course, the results of this study are subject to the dose-effect relationship. In the future, we will further clarify the respective advantages of *Astragalus* granules and chemically combined ingredients to provide new ideas and approaches for clinical use.

## 4. Conclusions

This study is based on network pharmacology and used the TCMSP, BATMAN, GeneCards, MalaCards, and OMIM databases to obtain AST and sepsis-related targets to identify shared genes between them. The AST could affect the TNF, PI3K, and MAPK pathway cascade responses centred on I*κ*B*α* and NF-*κ*B, attenuate the expression of IL-6 and MMP9, and interfere with the inflammatory response during sepsis.

## Figures and Tables

**Table 1:** Elution gradient.

Time (min)	%*A* (water)	%*B* (methanol)
0	80	20
8	0	100
13	0	100
15	80	20

**Table 2:** Specific primer sequences for GAPDH, *β*-actin, MAPK8, NF-*κ*B, I*κ*B*α*, PI3K, TNF-*α*, IL-6, and MMP9.

Gene	Primer	Sequence (5′-3′)	PCR products (bp)
Rat GAPDH	Forward	ACAGCAACAGGGTGGTGGAC	253
	Reverse	TTTGAGGGTGCAGCGAACTT	

Rat MAPK8	Forward	ATTTGGAGGAGCGAACTAAG	160
	Reverse	CTGCTGTCTGTATCCGAGGC	

Rat NF-*κ*B	Forward	TGACGGGAGGGGAAGAAATC	211
	Reverse	TGAACAAACACGGAAGCTGG	

Rat I*κ*B*α*	Forward	ATGGCTACCTGGGCATCGTG	136
	Reverse	TTCAACAGGAGCGAGACCAG	

Rat PI3K	Forward	GTGGTAGATGGCGAAGTCA	126
	Reverse	CAGGGAGGTGTGTTGGTAA	

Rat TNF-*α*	Forward	CCGATTTGCCATTTCATACCAG	232
	Reverse	TCACAGAGCAATGACTCCAAAG	

Rat IL-6	Forward	GTTGCCTTCTTGGGACTGATG	102
	Reverse	TACTGGTCTGTTGTGGGTGGT	

Rat MMP9	Forward	GCTGGGCTTAGATCATTCTTCAGTG	109
	Reverse	CAGATGCTGGATGCCTTTTATGTCG	

Rat *β*-actin	Forward	CACGATGGAGGGGCCGGACTCATC	240
	Reverse	TAAAGACCTCTATGCCAACACAGT	

**Table 3:** The chemical composition of AST and its targets.

Components	The number of target	Structural formula	Database
Mairin	1	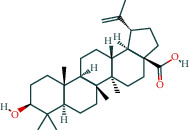	TCMSP
Jaranol	9	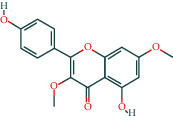	TCMSP
Kaempferol	51	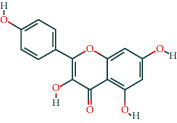	TCMSP
Isorhamnetin	24	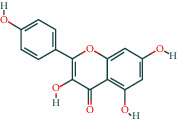	TCMSP
Bifendate	4	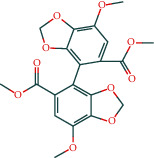	TCMSP
1,7-Dihydroxy-3,9-dimethoxy pterocarpene	3	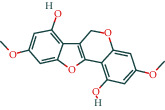	TCMSP
(6aR,11aR)-9,10-Dimethoxy-6a,11a-dihydro-6H-benzofurano[3,2-c]chromen-3-ol	18	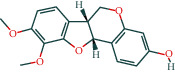	TCMSP
(3S,8S,9S,10R,13R,14S,17R)-10,13-Dimethyl-17-[(2R,5S)-5-propan-2-yloctan-2-yl]-2,3,4,7,8,9,11,12,14,15,16,17-dodecahydro-1H-cyclopenta[a]phenanthren-3-ol	1	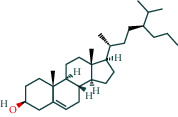	TCMSP
Tetradecane	98	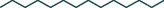	BATMAN
Tridecene	98	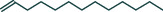	BATMAN
1,22-Docosanediol	159	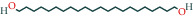	BATMAN
Ginsenoside	19	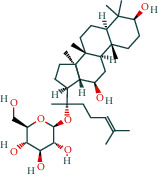	BATMAN
(-)-Dicentrine	47	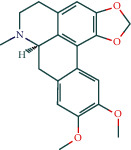	BATMAN
Notoginsenoside	1	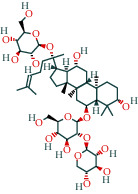	BATMAN
(-)2d,4d,6d,8d-Tetramethyl undecanoic acid	226	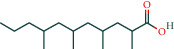	BATMAN
Sandaracopimarinol	51	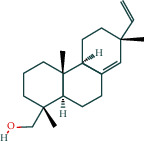	BATMAN
Ginsenoside Rb1	1	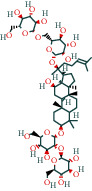	BATMAN
Dicapryl phthalate	13	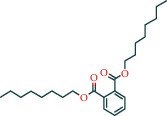	BATMAN
Cyperene	98	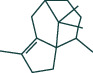	BATMAN
(-)-Trifara-9,14-diene	98	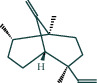	BATMAN
Cycloeucalenol	32	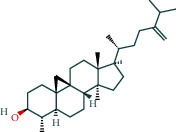	BATMAN
4,4′-Diketo-3-hydroxy-beta-carotene	43	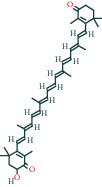	BATMAN
Nonadecanoic acid	22	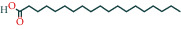	BATMAN
Quercetin	15	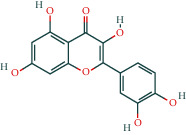	BATMAN
Notoginsenoside R3	1	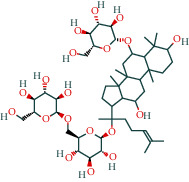	BATMAN
Ditertbutyl phthalate	27	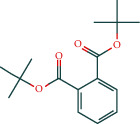	BATMAN
Hexadecanoic acid	226	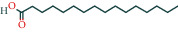	BATMAN
Dauricine	22	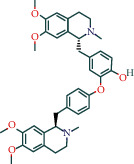	BATMAN
3,4-Dimethylbenzoic acid	131	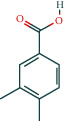	BATMAN
Notoginsenoside R1	1	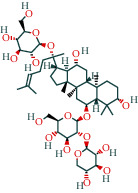	BATMAN
Notoginsenoside A	1	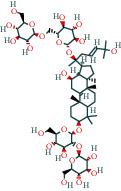	BATMAN
Gypenoside Xvii	1	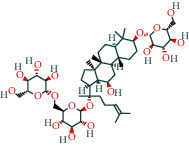	BATMAN
Epsilon-cadinene	98	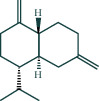	BATMAN
1-Tetradecanol	193	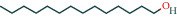	BATMAN
1-Heptadecanol	159	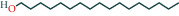	BATMAN
N-Nonanol	159	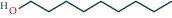	BATMAN
Acetophenone	40	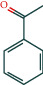	BATMAN
Encecalin	60	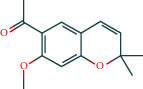	BATMAN
20-Hexadecanoylingenol	10	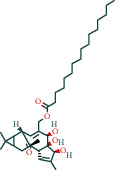	BATMAN
Notoginsenoside R2	1	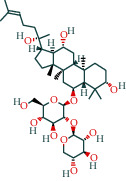	BATMAN
Elemicin	61	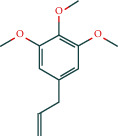	BATMAN
Sanchinoside B1	2	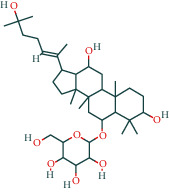	BATMAN
Alpha-cuparenol	4	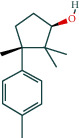	BATMAN
Cyclododecanone	23	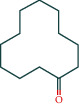	BATMAN
Stigmasterol	119	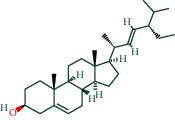	BATMAN
Panaxatriol	2	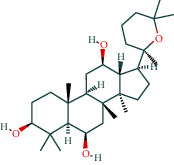	BATMAN
Panaxadiol	2	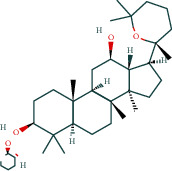	BATMAN
Heneicosanic acid	226	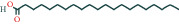	BATMAN
Coprine	386	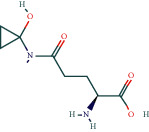	BATMAN
1,1,5,5-Tetramethyl-4-methano-2,3,4,6,7,10-hexahydronaphthalene	100	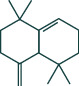	BATMAN
Delta-guaiene	98	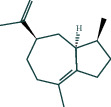	BATMAN
1-Methyl-4-isoallyl-cyclohexane	98	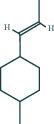	BATMAN
Gamma-sitosterol	35	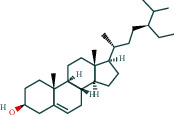	BATMAN
Pentadecanoic acid	226	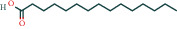	BATMAN
Ginsenoside-Rb2	1	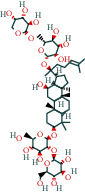	BATMAN
Cuparene	10	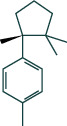	BATMAN
Ginsenoside-Rd	1	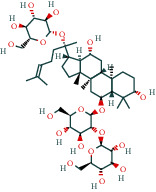	BATMAN
Delta-elemene	99	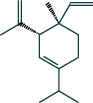	BATMAN
Cyperol	33	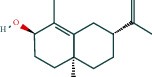	BATMAN

The table lists the names of the chemical components within *Astragalus*, the number of corresponding targets, their structural formulas, and the databases from which they are sourced.

## Data Availability

The data used to support the findings of the study are available from the corresponding author upon reasonable request.
